# Gastric Bypass Presenting as Mixed Beriberi and Wernicke Encephalopathy

**DOI:** 10.7759/cureus.103968

**Published:** 2026-02-20

**Authors:** Varshini Babu, Felicia D' Souza

**Affiliations:** 1 Internal Medicine, University of Pennsylvania, Philadelphia, USA

**Keywords:** beriberi, bypass, dry beriberi, gastric bypass surgery, thiamine deficiency, vitamin b1 deficiency, wernicke encephalopathy, wet beriberi

## Abstract

Thiamine deficiency can lead to a spectrum of neurologic and cardiovascular manifestations, most notably beriberi and Wernicke encephalopathy. This condition is further separated into wet (cardiovascular manifestations) and dry (neurological manifestations) beriberi. Beriberi is often caused by a prolonged mild to moderate thiamine deficiency. In contrast, Wernicke encephalopathy occurs due to a severe, short-term thiamine deficiency that can present as the classic triad of mental status changes, ocular abnormalities, and gait ataxia. Since both conditions are due to the same cause of thiamine deficiency, they can coexist, but this often makes diagnosis more difficult, especially when findings are more subtle. We describe a 46-year-old female with a history of Roux-en-Y gastric bypass surgery who was admitted for non-infectious colitis and subsequently new-onset right lower extremity weakness and numbness, altered mental status, hypotension, and tachycardia. Empiric IV thiamine was initiated based on clinical suspicion despite an otherwise reassuring workup. She was later confirmed to have severe thiamine deficiency. This case highlights the importance of having high suspicion for vitamin deficiencies despite nonclassical symptoms.

## Introduction

Thiamine deficiency can present as three clinical manifestations: wet beriberi, dry beriberi, and Wernicke encephalopathy. Wet beriberi, which primarily affects the cardiovascular system, is characterized by high-output heart failure due to elevated jugular venous pressure, peripheral edema, cardiac enlargement, and tachycardia [1]. Dry beriberi affects the nervous system, primarily causing peripheral polyneuropathy with symptoms worsening distally [2]. Dry beriberi can resemble polyradiculoneuropathy, similar to Guillain-Barré syndrome. Wernicke encephalopathy is an acute neurological disorder, often characterized by the classic triad of altered mental status, ataxia, and ocular abnormalities. However, only a minority of patients present with the complete triad of symptoms [3]. When Wernicke encephalopathy is not appropriately treated, it progresses to Wernicke-Korsakoff syndrome.

Risk factors for thiamine deficiency include malnutrition, bariatric surgery, gastrointestinal disorders, alcohol, and malignancy [4,5]. In well‑resourced settings, clinicians may be less likely to consider beriberi in the differential diagnosis, which can delay recognition. Because thiamine deficiency can produce overlapping cardiac and neurologic manifestations, clinicians should maintain a high index of suspicion in at‑risk patients and consider empiric thiamine when clinical concern is high.

This paper intends to demonstrate how some seemingly unrelated symptoms can be explained by a unifying diagnosis of thiamine deficiency, especially in a patient with prior psychiatric history and polypharmacy.

## Case presentation

A 46-year-old female with a history of Roux-en-Y gastric bypass (13 years prior) in the setting of morbid obesity, polypharmacy, and psychiatric history was admitted with an episode of acute non-infectious colitis that was conservatively managed with bowel rest, intravenous fluids, and pantoprazole. After 48 hours of admission, she started to slowly tolerate food intake.

On hospital day three, she developed new-onset distal right lower extremity weakness and numbness, blurry vision, lateral nystagmus, and slight tremors. She also became intermittently somnolent, requiring sternal rubbing and repeated prompting to wake up. Otherwise, her cranial nerve exam and reflexes were normal. A stroke alert was initiated, but computed tomography scan and magnetic resonance imaging (MRI) showed no evidence of acute infarct or hemorrhage. She was intermittently hypotensive to 90s/60s and tachycardic to 110s. On physical exam, her lower extremities demonstrated 1+ pitting edema bilaterally. An echocardiogram was only remarkable for mild aortic regurgitation (ejection fraction of 61%). Intermittent electrocardiograms demonstrated sinus tachycardia with no obvious arrhythmia. Thyroid testing and urine drug screen were unremarkable; lower extremity Dopplers excluded deep venous thrombosis. Labs were notable for chronic anemia, as well as hypoalbuminemia, hypocalcemia, and low urea nitrogen in the setting of poor intake with acute non-infectious colitis (Table 1).

**Table 1:** Serum laboratory findings.

Lab	Value	Reference range
Sodium (mmol/L)	144	136-144
Potassium (mmol/L)	3.7	3.6-5.1
Chloride (mmol/L)	113	101-111
Carbon dioxide (mmol/L)	25	22-32
Urea nitrogen (mg/dL)	3	8-20
Creatinine (mg/dL)	0.77	0.44-1.03
Calcium (mg/dL)	7.7	8.9-10.3
Magnesium (mg/dL)	2	1.8-2.5
Protein, total (g/dL)	5	6.1-7.9
Albumin (g/dL)	1.8	3.5-5.1
Bilirubin, total (mg/dL)	0.7	0.3-1.2
Alkaline phosphatase(U/L)	120	38-126
Aspartate aminotransferase (U/L)	41	15-41
Alanine aminotransferase (U/L)	34	14-54
WBC (10^3/uL)	4.5	4-11
Hemoglobin (g/dL)	11.1	12-16
Platelets (10^3/uL)	173	150-400

Given her unexplained episodes of somnolence, the patient’s sedating home medications, including alprazolam, hydroxyzine, and doxepin, were all discontinued in the following days. Moreover, the patient reported that pain and anxiety levels were well controlled. However, despite addressing these psychological and polypharmacy factors, the patient continued to report progressive, intermittent lower extremity weakness and numbness with a shift to worsening left-sided symptoms, in addition to continued intermittent somnolence.

**Figure 1:**
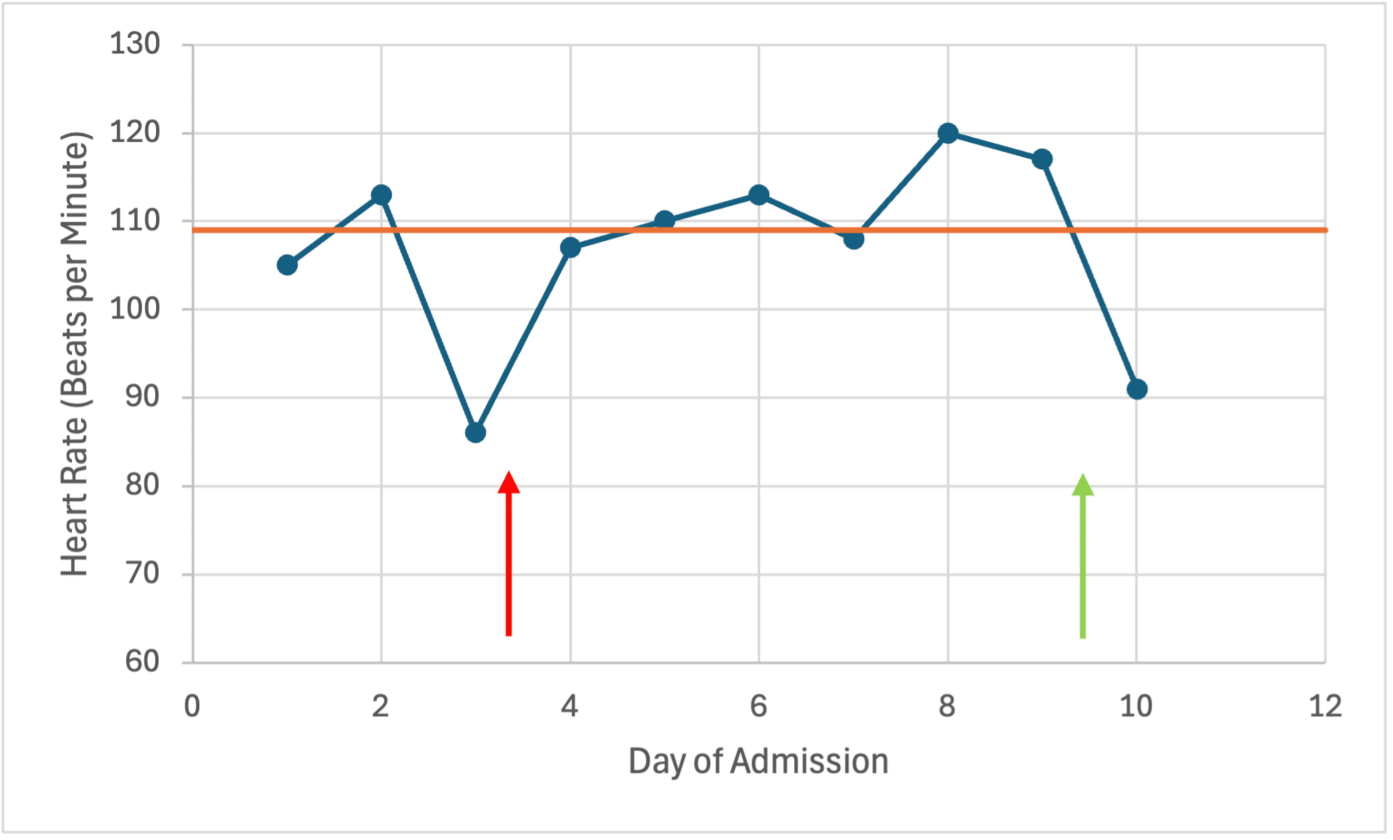
Patient's heart rate trend during admission with improvement of heart rate after thiamine administration. The red arrow indicates symptom onset, and the green arrow indicates thiamine administration. The orange line is the median heart rate at admission (109 beats per minute).

The patient had no prior history of alcohol use. However, given her numerous unexplained intermittent neurological and cardiovascular symptoms, and remote history of Roux-en-Y bypass, nutritional labs (thiamine, vitamin B12, folate, zinc, selenium, ceruloplasmin, vitamin D, vitamin A, vitamin E) were collected, and she was empirically started on thiamine 600 mg every eight hours intravenously. Folate levels and vitamin B12 levels were unremarkable. Within 24 hours of thiamine administration, the patient’s alertness and energy improved; tachycardia decreased to the 90s (Figure 1), and she started ambulating independently.

**Figure 2:**
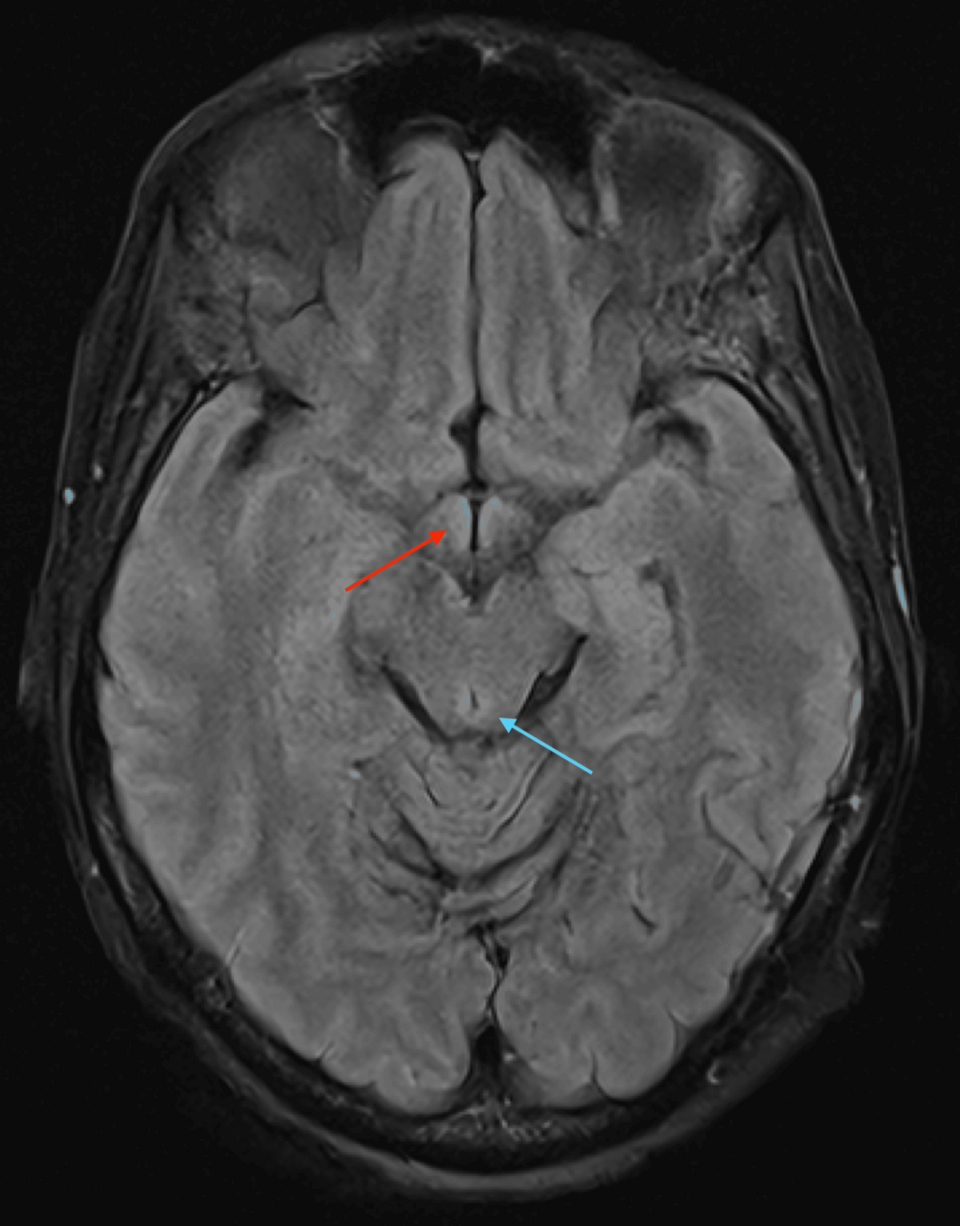
Subtle T2 FLAIR signal abnormalities, which may reflect developing magnetic resonance imaging findings. The red arrow indicates subtle hyperintensity in the mammillary bodies. The blue arrow indicates subtle hyperintensity in the periaqueductal gray matter. FLAIR: fluid-attenuated inversion recovery.

She was discharged to a facility on 100 mg thiamine tablets. The pre-treatment thiamine disphosphate level returned one week later at 35 nmol/L (normal: 70-180 nmol/L), confirming severe deficiency. Stroke MRI reviewed post diagnosis reflected some subtle changes that were correlated clinically (Figure 2).

## Discussion

This case illustrates post-bariatric surgery thiamine deficiency, a potentially life-threatening but preventable complication that can manifest with multisystem involvement, including neurological and cardiovascular symptoms [6]. The patient's presentation with non-infectious colitis, followed by intermittent neurological, cardiovascular, and gastrointestinal symptoms, combined with dramatic improvement after thiamine administration and confirmed low pre-treatment levels, is consistent with mixed beriberi and possible Wernicke's encephalopathy.

Thiamine deficiency after Roux-en-Y gastric bypass can occur through multiple mechanisms, including reduced gastric acid production that impairs thiamine absorption, decreased oral intake, malabsorption due to bypass of the duodenum and proximal jejunum, and recurrent vomiting [6]. Body thiamine stores last only 15-21 days, making patients vulnerable to rapid depletion during acute illness or poor intake [7]. In this case, the patient’s colitis and associated gastrointestinal symptoms likely precipitated acute-on-chronic thiamine deficiency, unmasking both mixed beriberi and Wernicke encephalopathy features.

The prevalence of thiamine deficiency after bariatric surgery is substantial, with 16-29% of post-bariatric patients experiencing vitamin B1 deficiency [8]. Polypharmacy and psychiatric history may also have contributed to poor nutritional compliance, a recognized risk factor for deficiency [3]. In this patient, these factors initially obscured the possibility of a vitamin deficiency because they could plausibly explain several neurological symptoms.

The multisystem presentation of this case reflects the variety of pathophysiological derangements that can be caused by thiamine. In a systematic review of post-bariatric surgery patients with clinical thiamine deficiency, 70% had cardiac manifestations, 59% had peripheral neurological symptoms, 14% had gastrointestinal symptoms, and 5% had neuropsychiatric manifestations [9].

Wernicke's encephalopathy occurs most commonly within six months after bariatric surgery, with 94% of cases developing in this timeframe [7]. However, thiamine deficiency can occur years after bariatric surgery, especially when exacerbated by an acute stressor, as had occurred in this case. The classic triad of confusion, eye movement abnormalities, and gait instability is often incomplete, making diagnosis challenging [7]. This patient's intermittent neurological symptoms, even if not presenting as the classic triad, warranted empiric thiamine treatment given the high-risk context.

The dramatic response to thiamine administration confirms the diagnosis and underscores the importance of prompt recognition and empiric treatment in high-risk patients. For suspected Wernicke's encephalopathy, immediate high-dose thiamine (500 mg three times daily intravenously) should be administered without waiting for laboratory confirmation, especially because laboratory testing of thiamine often takes multiple days [10]. Clinical improvement typically begins within 24-48 hours of thiamine administration in patients with thiamine deficiency, as demonstrated by this patient's course [11]. Suboptimal thiamine dosing can lead to residual cognitive deficits with potential for progression to Wernicke-Korsakoff’s syndrome [3]. It is recommended that all post-bariatric surgery patients take at least 12 mg of thiamine daily, with 50-100 mg daily from B-complex supplements potentially needed to maintain adequate levels [12].

This case highlights several critical points: (1) thiamine deficiency can occur years after bariatric surgery; (2) acute illness such as colitis can exacerbate symptomatic deficiency in patients with subclinical depletion; (3) presentation may be atypical or involve multiple organ systems; and (4) vitamin deficiency should always be considered even when the patient has competing explanations, including history of psychiatric illness and polypharmacy.

## Conclusions

Thiamine deficiency is a dangerous vitamin deficiency that can be fatal if missed. This case demonstrates that the symptoms of thiamine deficiencies, including beriberi and Wernicke encephalopathy, often overlap. This case also showed the importance of prompt recognition and empiric treatment in high-risk patients. Since thiamine laboratory testing often takes multiple days, it is essential for healthcare professionals, especially those from well-resourced countries, who are not regularly exposed to vitamin deficiency cases, to be aware of how the nonspecific symptoms of different thiamine deficiencies can present together.
